# Rare Hypothenar Myxoma Causing Ulnar Neuropathy: Histopathology and Treatment Pearls

**DOI:** 10.1097/GOX.0000000000001806

**Published:** 2018-07-13

**Authors:** Peter K. Firouzbakht, Jacqueline S. Israel, Brian M. Christie, Venkat K. Rao

**Affiliations:** From the * Division of Plastic Surgery, Department of Surgery, University of Wisconsin School of Medicine and Public Health, Madison, Wis.; † Texas Tech University Health Science Center School of Medicine, Lubbock, Tex.

## Abstract

A myxoma is a neoplasm comprised of mesenchymal connective tissue. Myxomas of the upper extremity, and particularly of the hand, are rare. We present a case of a hypothenar myxoma causing ulnar neuropathy in a patient with a history of acute inflammatory demyelinating process. Treatment and management of myxoma may vary depending on whether the process is malignant or benign; thus, histologic diagnosis is critical to determining treatment. The purpose of this article is to review the pathophysiology and clinical features of myxomas, and to provide recommendations for evaluating and treating individuals with extremity masses of unclear clinical diagnosis.

## CASE

**Fig. 1.**
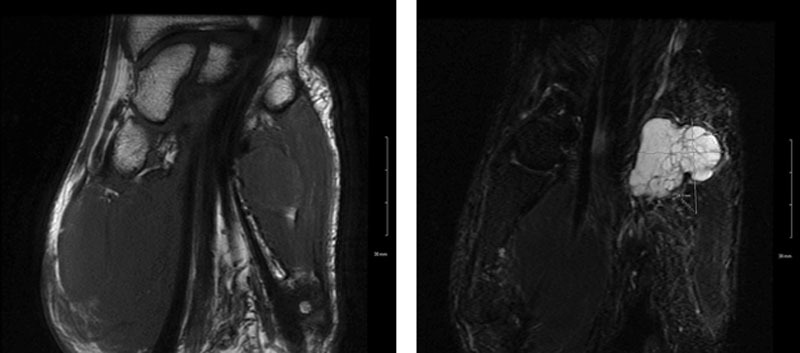
Magnetic resonance angiography images of the left hand. Coronal T1 (left) and coronal STIR (right) images reveal a 2.6 x 2.6 x 2.9 cm lobulated, multiseptated mass in the hypothenar eminence centered over the hook of hamate. The mass demonstrates T2 hyperintensity with internal T2 dark septa, T1 isointensity to muscle, and thick, irregular peripheral enhancement. There is no surrounding tissue edema and no additional masses are identified. Based on imaging findings, the differential diagnosis included myxoma, soft tissue sarcoma, cystic lesion, and malignant peripheral nerve sheath tumor.

A 55-year-old right-hand-dominant male with a history of acute inflammatory demyelinating process and a 3-month history of a left volar wrist and hypothenar soft tissue mass presented for evaluation. The patient was admitted to the hospital with generalized weakness, ataxia, and multiple sensory deficits, including complete lack of sensation in the left small finger and ulnar side of his hand. Although his sensory deficits were initially thought to relate to his generalized demyelinating disorder, on examination, the ulnar deficit was thought to be related to the mass in his hand. The mass was soft, nontender, and did not limit range of motion. Two-point sensation was absent in the ulnar nerve distribution distal to the wrist, and grip strength, while not measured objectively, was decreased compared with the contralateral side. There was no history of trauma, previous surgery, or other masses, and radiographs of the left upper extremity were normal. A lipoma was the suspected initial diagnosis. Magnetic resonance angiography of the left hand showed a soft-tissue mass of unclear etiology (Fig. 1).

**Fig. 2.**
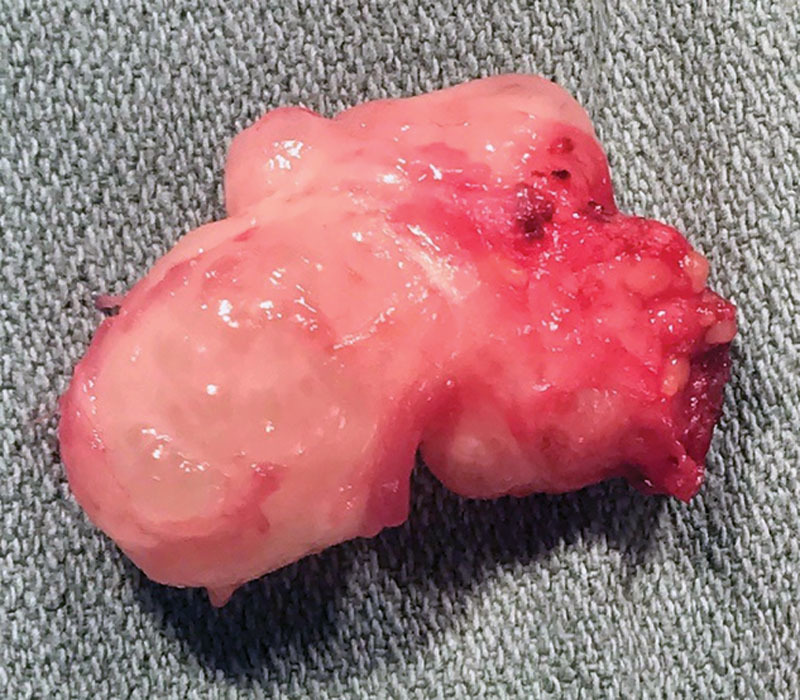
Intraoperative photograph of the hypothenar mass after exploration and excision. Grossly, the mass was found be tan-white in color with a rubbery, lobulated consistency.

**Fig. 3.**
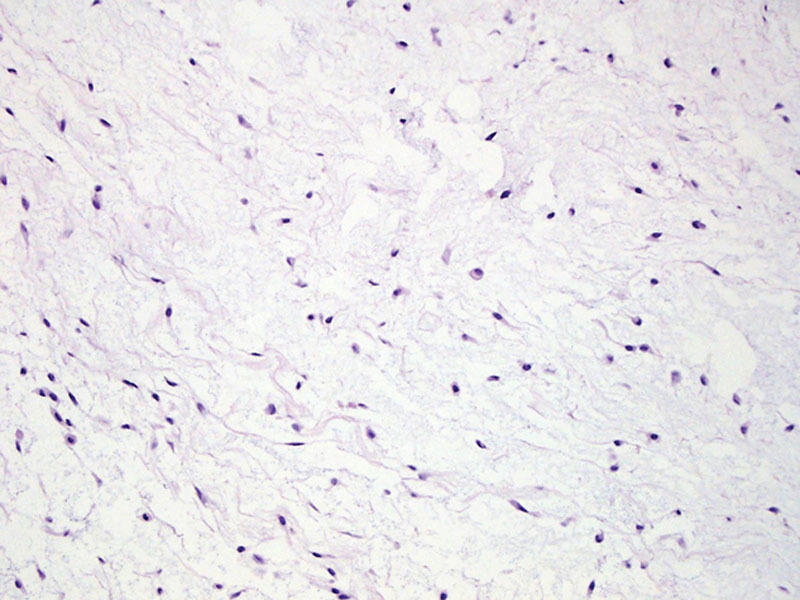
Histopathologic assessment at 100x magnification revealed relatively monotonous spindled to stellate cells with minimal pleomorphism and no appreciable mitotic activity (100x). These histologic findings were consistent with soft tissue myxoma.

Operative excision was performed under general anesthesia. A longitudinally oriented ulnar-sided skin incision was made over the mass, with dissection through the palmar fascia. The ulnar nerve and artery were dissected free from the mass, and Guyon’s canal was released. The mass was noted to be arising from beneath the hypothenar musculature. Grossly, it was tan-white, rubbery, and lobulated (Fig. 2). Pathology confirmed the mass to be a benign myxoma (Fig. 3). There were no postoperative complications. At 5 weeks postoperatively, the patient reported marked improvement in his numbness and weakness. He was found to have intact sensation in the ulnar nerve distribution distal to the site of the excised mass, and improved grip strength. The patient was asked to return in several months for repeat sensorimotor assessment, but was subsequently lost to follow-up.

## DISCUSSION

Soft-tissue myxoma was first described in 1863 by Virchow as a benign tumor similar to mucous tissue of the umbilical cord.^1,2^ Stout’s 1948 definition refers to myxoma as, “a neoplasm composed of stellate cells set in a loose mucoid stroma through which course very delicate reticulin fibers in various directions.”^3^ While the histologic criteria of myxoma are well-established, the etiology remains unclear.^1^ Although the majority of myxomas occur in the heart, extracardiac myxomas do occur, and may be found either subcutaneously or embedded within muscle; intramuscular myxomas are generally found in the thigh, lower leg, or gluteal region, with an incidence of approximately 0.1 per 100,000.^1,4–7^

Myxomas of the hand are rare and may be confused with other benign and malignant neoplasms on clinical examination or after imaging.^5,6^ Myxomas have been reported to occur in the fingertip, phalanges, and metacarpal and palmar regions.^1,6^ Benign myxomas of the upper limb are classified according to the tissue of origin, with subungual myxomas occurring most commonly, and intraosseous and intramuscular myxomas occurring most rarely.^4,8^

We report a case in which the patient’s history of an acute demyelinating disease made diagnosis more challenging. Without knowledge of the hypothenar myxoma, the symptoms of hand numbness and weakness had been attributed to acute inflammatory demyelinating process. However, after a thorough hand examination, discovery of the mass and patient’s ulnar neuropathy supported the decision to excise the symptomatic soft-tissue mass.

Magnetic resonance imaging allows for characterization of soft-tissue lesions of the hand and should be used when a myxoma is suspected to aid ascertainment of whether the mass arises from a nerve sheath or has an intramuscular course.^1^ However, myxoma may be confused with other types of lesions, and other etiologies such as sarcoma, cysts, and infectious processes must not be ruled out until histopathologic information can confirm the diagnosis.^1^

Myxomas are most frequently benign, though they may infiltrate surrounding tissues and structures.^1,4,6^ Malignant myxomas (eg, myxosarcoma) display higher vascularity, infiltration, and more variation in thickness when silver-stained than their benign counterparts.^9^ If a biopsy is performed, a myxoma should be excised, regardless of malignancy or presence/absence of symptoms.^1,4,6^ Some authors report obtaining a diagnosis using an intraoperative frozen section.^1,4^ Recurrence is generally rare and may be associated with incomplete resection or malignancy.^1,4,9^ Malignant myxomas are capable of metastasizing^9^; thus, subsequent treatment is different than local excision of a benign myxoma, often requiring more radical excision and, in some cases, amputation.^9^

## CONCLUSIONS

Myxomas of the upper extremity, particularly the hand, are rare. Although they are generally benign, malignant transformation has been described and should not be overlooked in a differential diagnosis. Resection is recommended, with close attention to whether the mass involves and/or arises from neurovascular structures.

## ACKNOWLEDGMENTS

The authors greatly appreciate pathologists Michael L. Schwalbe, MD, and Thomas Warner, MD.
